# A pilot study of change in fracture risk in patients with acute respiratory distress syndrome

**DOI:** 10.1186/s13054-015-0892-y

**Published:** 2015-04-14

**Authors:** Jaikitry Rawal, Mark JW McPhail, Gamumu Ratnayake, Pearl Chan, John Moxham, Stephen DR Harridge, Nicholas Hart, Hugh E Montgomery, Zudin A Puthucheary

**Affiliations:** Institute of Health and Human Performance, University College London, Room 443, 74 Huntley Street, London, WC1E 6AU UK; Department of Hepatology and Gastroenterology, St Mary’s Hospital, Imperial College London, praed street, London, W2 1NY UK; Institute of Liver Studies, King’s College Hospital NHS Foundation Trust, denmark hill, London, SE59RS UK; NIHR Comprehensive Biomedical Research Centre, Guy’s and St Thomas’ NHS Foundation Trust and King’s College London, Westminster Bridge Road, London, SE17EH UK; King’s College London School of Medicine, denmark hill, London, SE59RS UK; Centre of Human and Aerospace Physiological Sciences, King’s College London, Great Maze Pond, London, SE1 9RT UK; Division of Respiratory and Critical Care, National University Hospital, 1E Lower Kent Ridge Road, Singapore, 119228 Singapore

## Abstract

**Introduction:**

Acute skeletal muscle wasting is a major contributor to post critical illness physical impairment. However, the bone response remains uncharacterized. We prospectively investigated the early changes in bone mineral density (BMD) and fracture risk in critical illness.

**Methods:**

Patients were prospectively recruited ≤24 hours following intensive care unit (ICU) admission to a university teaching or a community hospital (August 2009 to April 2011). All were aged >18 years and expected to be intubated for >48 hours, spend >7 days in critical care and survive ICU admission. Forty-six patients were studied (55.3% male), with a mean age of 54.4 years (95% confidence interval (CI): 49.1 to 59.6) and an APACHE II score of 23.9 (95% CI: 22.4 to 25.5). Calcaneal dual X-ray absorptiometry (DXA) assessment of BMD was performed on day 1 and 10. Increase in fracture risk was calculated from the change in T-score.

**Results:**

BMD did not change between day 1 and 10 in the cohort overall (0.434 (95% CI: 0.405 to 0.463) versus 0.425 g/cm^2^ (95% CI: 0.399 to 0.450), *P* = 0.58). Multivariable logistical regression revealed admission corrected calcium (odds ratio (OR): 1.980 (95% CI: 1.089 to 3.609), *P* = 0.026) and admission PaO_2_-to-FiO_2_ ratio (OR: 0.916 (95% CI: 0.833 to 0.998), *P* = 0.044) to be associated with >2% loss of BMD. Patients with acute respiratory distress syndrome had a greater loss in BMD than those without (−2.81% (95% CI: −5.73 to 0.118%), n = 34 versus 2.40% (95% CI: 0.204 to 4.586%), n = 12, *P* = 0.029). In the 34 patients with acute respiratory distress syndrome, fracture risk increased by 19.4% (95% CI: 13.9 to 25.0%).

**Conclusions:**

Patients with acute respiratory distress syndrome demonstrated early and rapid bone demineralisation with associated increase in fracture risk.

**Electronic supplementary material:**

The online version of this article (doi:10.1186/s13054-015-0892-y) contains supplementary material, which is available to authorized users.

## Introduction

Rapid and early muscle wasting contributes to the significant long-term functional impairment observed in survivors of critical illness [1-4]. Although muscle and bone mass correlate in healthy individuals [5-7], there are limited data reporting the impact of critical illness on bone health. During acute critical illness, mechanical unloading as a consequence of bed rest [8], inflammation [9], acidaemia [10], vitamin D deficiency [11], corticosteroid use [12] and hypoxia [13] may all worsen bone health and reduce bone mineral density (BMD). Indeed, markers of bone turnover increase during critical illness [11,14]. Such bone demineralization may explain symptoms of generalised musculoskeletal pain in survivors of critical illness [1,2], and the reported increase in fracture risk in elderly females following critical illness [15]. However, the bone response to critical illness has never before been prospectively studied. We thus performed a pilot study to investigate the early effects of critical illness on BMD and fracture risk, and also sought clinical factors that might be associated with early bone demineralization.

## Materials and methods

Subjects comprised a subgroup of the Musculoskeletal Ultrasound in Critical Illness: Longitudinal Evaluation cohort (trial registered with Clinicaltrials.gov, identifier: NCT01106300) [4]. Ethical approval was obtained from the University College London ethics committee. Patients were recruited within 24 hours of admission to a university hospital (Kings College Hospital NHS Foundation Trust) or a community hospital intensive (Whittington Hospital NHS Trust) care unit (ICU) between August 2009 and April 2011. All were anticipated to be invasively ventilated for more than 48 hours, spend more than 7 days in the ICU and survive their ICU stay. Patients who were pregnant or suffering lower limb amputation, primary neuromuscular disease or cancer were excluded. At enrolment, written assent was obtained from the next-of-kin, with retrospective patient consent obtained when full mental capacity was regained.

BMD was assessed using dual X-ray absorptiometry (DXA) imaging on day 1 and day 10 (DXL Calscan, Demetech AB, Sweden), which has a coefficient of variance of 0.9%. Fracture risk was calculated from a change in T-score whereby the relative risk of a major osteoporotic fracture increases 1.5-fold (95% confidence interval (CI): 1.4 to 1.6) per standard deviation below the mean T-score [16]. Detailed clinical and physiological bedside data were collected, as previously described [4].

### Statistical analysis

All data were assessed for normality using D’Agostino and Pearson omnibus normality tests, and analyzed using Student’s t-test, Pearson’s coefficient, Mann-Whitney U test and Wilcoxon’s signed rank tests, as appropriate. For the purposes of hypothesis generation in this pilot study, we sought a parsimonious model of associated physiological factors. Age, sex and chronic disease were forced into bivariable logistical regression (Statistical Package for the Social Sciences version 17; SPSS Inc., Chicago, IL, USA) using a threshold of 2% loss of BMD, which is twice the expected loss from bed rest alone [8]. Statistically significant independent variables from the bivariable analysis were entered into a backward multivariable analysis if the *P* value was 0.10 or less.

## Results

Fifty-seven patients assented to serial DXA scanning. Of these, seven did not survive ten days, one was transferred to another hospital, one withdrew from the study, one was discharged before day ten and one was unable to have serial scans for technical reasons. Forty-six patients were included in the final analysis. The characteristics of these 46 patients, shown in Table 1, did not differ from those withdrawn, except for a higher Simplified Acute Physiology Score (SAPS II) (40.9 (95% CI: 37.5 to 44.3, n = 46) versus 53.3 (95% CI: 44.2 to 62.4, n = 10); *P* <0.01). Four patients had pre-morbid conditions associated with possible disrupted calcium homeostasis (one with hypothyroidism, one with Crohn’s disease, two with hyperthyroidism). Their baseline DXA measurement was no different from the remaining cohort (0.387 ± 0.07 versus 0.437 ± 0.01, *P* = 0.350). Twenty eight patients (61%) were defined as having osteopenia on day one of the study; however, no patients received renal replacement therapy using citrate anticoagulation with calcium replacement, and no subjects received regular selective serotonin reuptake inhibitors or serotonin norepinephrine reuptake inhibitors (associated with lower BMD) prior to critical illness. Further data regarding recruitment and survival, as well as baseline laboratory values, are available in Additional file 1.

**Table 1:** **Characteristics of patients who had serial measurements versus those who only had admission measurements**

	**Serial DEXA measurements**	**Single DEXA measurement**	***P*** **value**
N	46	11	-
Age	55.09 (49.9-60.3)	53.9 (40.6-67.1)	0.93
Male sex, n (%)^b^	26 (55.3)	6 (60)	0.74
Pre-ICU LOS^a^	1 (1-45)	1 (1-6)	0.25
APACHE II score	24 (22.4-25.6)	27.1 (21.9-32.3)	0.12
SAPS II score	40.9 (37.5-44.3)	53.3 (44.2-62.4)	<0.01^c^
Admission SOFA score	9.3 (8.5-10.0)	8.6 (5.8-11.4)	0.50
Admission diagnosis, n (%)			
Cardiogenic shock	6 (13.0)	3 (33.3)	
Trauma	12 (26.1)	2 (20.0)	
Acute renal failure	1 (2.2)	2 (20.0)	
Intra cranial haemorrhage	5 (10.9)	1 (10.0)	
Acute liver failure	4 (8.7)	1 (10.0)	
Severe sepsis	16 (34.8)	1 (10.0)	
Major haemorrhage	2 (4.3)	0 (0.0)	
Co-morbidities, n (%)			
Ischaemic heart disease	5 (10.9)	3 (33.3)	
Liver cirrhosis	6 (13.0)	0 (0.0)	
Haematological disease	2 (4.3)	1 (10.0)	
Hypertension	9 (19.6)	1 (10.0)	
Obesity	2 (4.3)	0 (0.0)	
COPD	7 (15.2)	0 (0.0)	
Diabetes mellitus	5 (10.9)	0 (0.0)	
Previous CVA	1 (2.2)	0 (0.0)	
Chronic pancreatitis	1 (2.2)	0 (0.0)	
Thyroid disease	3 (6.5)	0 (0.0)	
Crohn’s disease	1 (2.2)	0 (0.0)	
Renal impairment	2 (4.3)	0 (0.0)	
Small bowel insufficiency	0 (0.0)	1 (10.0)	

APACHE II = Acute Physiology and Chronic Health Evaluation II, COPD = Chronic Obstructive Pulmonary Disease, CVA = Cerebro Vascular Accident, DEXA = Dual X-ray Absorptiometry, ICU = Intensive Care Unit, LOS = Length of Stay, SAPS II = Simplified Acute Physiology Score, SOFA = Sequential Organ Failure Assessment. Values are mean (95% confidence intervals), except for ^a^indicating median with range. Student’s T-test was used except for ^b^(chi-squared) and ^a^(Mann Whitney U);^c^ indicates *P* <0.05.

### Clinical associations with change in bone mass density

BMD data were non-normally distributed prior to and following log transformation. There was no change in BMD between day 1 and 10 in the cohort overall (0.434 (95% CI: 0.405 to 0.463) versus 0.425 g/cm^2^ (95% CI: 0.399 to 0.450), *P* = 0.58). Multivariable logistical regression, adjusted for age, sex and chronic disease, was performed using a threshold of 2% loss of BMD. The model (overall model fit *P* <0.001, Hosmer-Lemeshow test *P* = 0.90, Table 2) demonstrated that admission calcium corrected for serum albumin (odds ratio (OR): 1.980 (95% CI: 1.089 to 3.609), *P* = 0.026) and the ratio of arterial partial pressure of oxygen (PaO_2_) to fraction of inspired oxygen (FiO_2_) on admission (OR: 0.916 (95% CI: 0.833 to 0.998), *P* = 0.044) were associated with a greater than 2% loss of BMD. Patient with acute respiratory distress syndrome (ARDS) (PaO_2-_to-FiO_2_ ratio of less than 300 mmHg [17] demonstrated greater BMD loss than those without (−2.81% (95% CI: −5.73 to 0.118%), n = 34 versus 2.40% (95% CI: 0.204 to 4.586%), n = 12, *P* = 0.029).

**Table 2:** **Bivariable and multivariable logistical analysis of bedside physiology versus 2% loss of bone mineral density by day 10**

		**Univariate**			**Multivariate**	
**Variable**	**OR**	**95% CI**	***P*** **value**	**OR**	**95% CI**	***P*** **value**
Admission BMD	1.000	0.997-1.003	0.899			
Change in RF_CSA_	1.019	0.971-1.070	0.439			
Organ failure	1.078	1.005-1.157	**0.037** ^**g**^			
Age	0.980	0.946-1.015	0.268	0.977	0.939-1.059	0.935
CRP^a^	1.000	0.999-1.001	0.857			
Chronic disease^e^	1.385	0.417-4.602	0.595	1.266	0.167-9.596	0.819
Insulin^a,b^	1.046	0.937-1.167	0.421			
Protein^a,b^	1.034	0.941-1.136	0.491			
Calories^a,b^	1.007	0.996-1.017	0.220			
LMWH^f^	1.001	0.998-1.004	0.530			
Unfractionated heparin^f^	1.000	1.000-1.000	0.518			
All heparin^f^	1.000	1.000-1.000	0.725			
Male sex	1.711	0.499-5.871	0.393	4.966	0.567-43.567	0.147
APACHE II	1.050	0.937-1.176	0.403			
SAPS II	0.995	0.944-1.050	0.867			
Admission SOFA	1.080	0.846-1.381	0.536			
Temperature	0.788	0.489-1.270	0.328			
Haemoglobin	1.047	0.794-1.381	0.744			
White cell count	0.886	0.778-1.009	**0.068**			
Platelets	0.994	0.988-1.000	**0.053**			
INR	4.960	0.886-27.774	**0.068**			
APTTR	2.830	0.403-19.873	0.296			
Sodium	0.988	0.871-1.121	0.856			
Potassium	0.475	0.160-1.411	0.180			
Urea	0.999	0.891-1.121	0.992			
Creatinine	1.001	0.990-1.011	0.914			
Alkaline phosphatase	1.010	0.995-1.025	0.178			
AST	1.003	0.999-1.006	**0.097**			
Bilirubin	1.048	0.998-1.101	**0.061**	1.078	0.994-1.156	0.076
Albumin	1.027	0.944-1.117	0.539			
**Calcium** ^**g**^	**1.974**	**1.116-3.492**	**0.020** ^**g**^	**1.980**	**1.089-3.609**	**0.026**
Phosphate	0.653	0.195-2.191	0.491			
Magnesium	0.435	0.036-5.190	0.510			
PaO_2_	0.836	0.659-1.061	0.141			
SaO_2_	0.840	0.617-1.145	0.271			
PaCO_2_	0.943	0.600-1.482	0.798			
H^+^	1.000	0.993-1.006	0.960			
Base excess	0.967	0.812-1.151	0.706			
Bicarbonate	1.024	0.839-1.249	0.815			
Lactate	1.018	0.669-1.549	0.933			
Chloride	1.047	.0935-1.172	0.424			
Apparent SID	0.914	.0792-1.055	0.218			
Effective SID	0.641	0.933-1.118	0.641			
Strong ion gap	0.968	0.905-1.035	0.338			
Glucose day 1	0.923	0.622-1.370	0.692			
MAP	1.010	0.947-1.077	0.768			
Heart rate	0.994	0.960-1.029	0.735			
**PaO**_**2**_ **/FiO**_**2**_ **ratio**	**0.943**	**0.890-1.000**	**0.050** ^**g**^	**0.916**	**0.833-0.998**	**0.044**
SIRS	0.544	0.154-1.925	0.345			
NMB use	1.154	0.742-1.795	0.524			
Corticosteroid use^c^	1.000	1.000-1.001	0.856			
RRT	1.094	0.291-4.109	0.894			
HMGCoA use^d^	2.000	0.483-8.275	0.339			
Median glucose^a^	1.158	0.637-2.104	0.631			
Days of intubation	0.988	0.923-1.057	0.724			
LOS pre-admission	1.166	0.681-1.994	0.576			

Organ failure was defined by SOFA scoring. All values are for day 1 of ICU admission, except ^a^which denotes area under curve for 10 days. ^b^Indicates those normalised to ideal body weight. ^c^Corticosteroid doses calculated in hydrocortisone equivalents. ^d^Denotes use on admission, and continued through study period. ^e^Chronic disease defined by hospital and general practice coding for management of chronic disease. ^f^Indicates cumulative dose. ^g^calcium variable, exponentially transformed to allow logistic regression. Bold type indicates *P* <0.05.

APACHE II = Acute Physiology and Chronic Health Evaluation II score, APTTR = Activated partial thromboplastin time ratio, AST = Aspartate transaminase, CRP = C-reactive protein, FiO_2_ = Fraction of inspired oxygen, HMGCoA RI = 3-hydroxy-3-methyl-glutaryl-CoA reductase inhibitor treatment, INR = International normalised ratio, LMWH = Low molecular weight heparin, LOS = Length of stay, NMB = Neuromuscular blockade, PaCO_2_ = Partial pressure of carbon dioxide in arterial blood, PaO_2_ = Partial pressure of oxygen in arterial blood, RF_CSA_ = Rectus Femoris cross-sectional area in 10 days, RRT = Renal replacement therapy, SaO_2_ = Oxygen saturation in arterial blood, SAPS 2 = Simplified Acute Physiology Score 2, SID = Strong Ion Difference, SOFA = Sequential Organ Failure Assessment.

### Change in T-score

**Figure 1:**
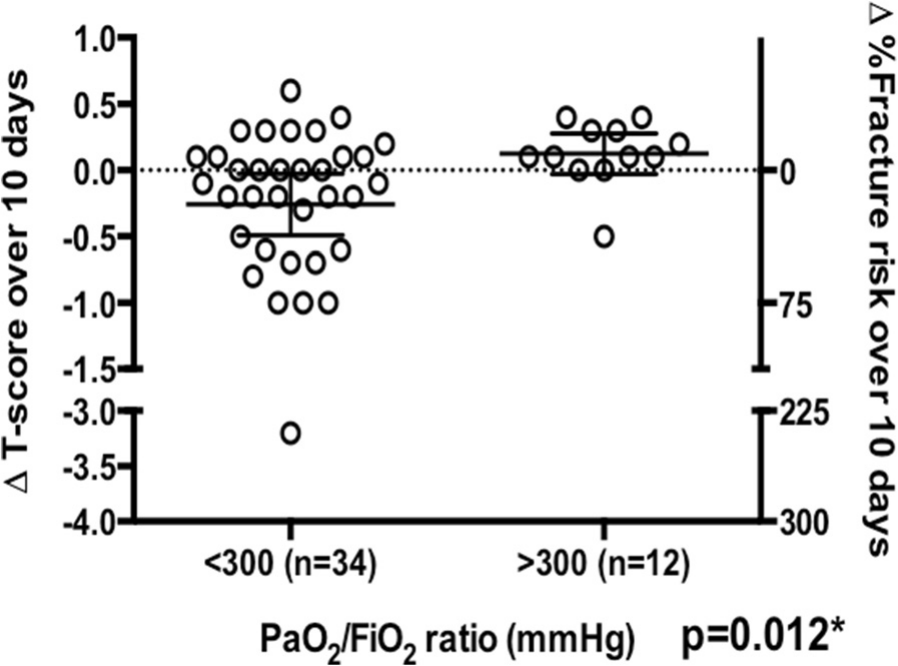
Change in T-score and percentage increase in fracture risk in patients with (n = 34) and without (n = 12) acute respiratory distress syndrome. FiO_2_ = Fraction of inspired oxygen, PaO_2_ = Partial pressure of oxygen in blood. Mann-Whitney U test was performed between groups, *P <0.05.

Admission T-score in those with ARDS did not differ from those without (−1.259 (95% CI: −1.737 to −0.781), n = 34 versus −1.325 (95% CI: −1.969 to −0.681), n = 12, *P* = 0.617), but ARDS patients had a change in T-score from day 1 to day 10 (−1.259 (95% CI: −1.737 to −0.781), n = 34 versus −1.518 (95% CI: −1.922 to −1.114), *P* = 0.047) compared to those without (−1.325 (95% CI: −1.969 to −0.681) versus −1.200 (95% CI: −1.798 to −0.602), *P* = 0.101). In the 34 patients with ARDS, fracture risk increased by 19.4% (95% CI: 13.9 to 25.0%) in the first 10 days of critical illness in comparison to those without (9.35% (95% CI: −2.1 to 20.9%), *P* = 0.012, Figure 1).

## Discussion

### Clinical relevance

The relationship between loss of BMD and rise in serum calcium concentration is to be expected. However, rapid early bone demineralization, of similar magnitude to that observed after much more prolonged weightlessness in space [18], occurs rapidly and early in critically ill patients with ARDS. The association of ARDS with bone demineralization is physiologically plausible: ARDS results in systemic release of inflammatory cytokines [19] such as interleukin-6 [20], TNFα [20,21], interleukin-1 [21] and interleukin-8 [22], which stimulate osteoclastogenesis and bone resorption with calcium mobilized into the circulation from these bone stores [23]. The scale of this loss is associated with an increase in calculated prospective fracture risk; an issue of some importance given that ARDS survivors have a number of independent risk factors for falls [24], including accelerated skeletal muscle wasting [4] and marked loss of executive function [25]. Bone is certainly able to respond rapidly to remodelling forces: changes are observed within 15 to 21 days in rodent models of unloading [26,27], and markers of bone turnover alter with single bouts of exercise in humans [28]. However, we are unaware of studies of the human skeletal response to illness over timeframes a short as that which we have addressed. Whilst *in vitro* evidence exists that hypoxia is detrimental to skeletal physiology and has both an inhibitory effect on osteoblastogenesis [29] and an activator-enhancing effect on osteoclastogenesis [30], no relationship was seen with admission hypoxia, although intermittent hypoxia as a stimulus cannot be excluded.

### Limitations

Whilst acknowledging that the first day of ICU admission is not the first day of critical illness, the median time to ICU admission was 24 hours, and 23 patients were admitted after a sudden acute event (for example trauma, myocardial infarction or intracranial bleed) with no antecedent decline. Nonetheless, these data should be considered hypothesis-generating. The limited sample size also precludes detailed exploration of other risk factors, such as osteopaenia, and generalisation to specific patient groups. Regrettably, we are unable to determine whether the observed changes translated into clinical skeletal events, given that this pilot study was not funded for the post-discharge follow-up of patients. Larger observational cohort studies, with extended follow-up periods, are required to determine the impact of critical illness on BMD and actual fracture risk. The determination of calcaneal BMD using DXA is valid and comparable to hip and spine DXA in determining fracture risk [16,31], and is more readily performed than the assessment of spine and hip bone densitometry in a remote imaging facility during the early stage of critical illness when the patient is most unstable. The coefficient of variance of DXA measures was 0.9%. A loss of more than 2% in calcaneal BMD (as we sought) was thus likely to represent true loss as opposed to measurement error.

## Conclusions

Rapid bone demineralization, associated with an increase in fracture risk, was observed in critically ill patients with ARDS. More extensive and extended hypothesis-driven epidemiological cohort studies are required to confirm this finding, and to determine whether bone demineralisation represents a new therapeutic target in reducing morbidity following critical illness.

## Key messages

- Loss of bone mineral density occurs rapidly in critically ill patients with acute respiratory distress syndrome.
- This is likely to be associated with an increase in fracture risk.

## Additional file

Additional file 1:
**Supplementary data.** 1.1 Baseline laboratory data. 1.2 Flowchart of patient recruitment and survival within study.

## Abbreviations

ARDS: Acute Respiratory Distress Syndrome
BMD: Bone Mineral Density
CI: Confidence interval
DXA: Dual X-ray Absorptiometry
FiO_2_: Fraction of inspired oxygen
ICU: Intensive Care Unit
OR: Odds ratio
PaO_2_: Arterial partial pressure of oxygen
SAPS II: Simplified acute physiology score
TNFα: tumor necrosis factor alpha
