# Supplementary material for: A pilot study of change in fracture risk in patients with acute respiratory distress syndrome
Source: Crit Care. 2015 Apr 14;19(1):165. doi: 10.1186/s13054-015-0892-y (PMC4411936; doi:10.1186/s13054-015-0892-y)

***Rapid change in fracture risk in patients with acute lung injury-online supplement***

- 1. ***Baseline laboratory values***

| Laboratory variable | Admission mean (95%CI) |
| --- | --- |
| Haemoglobin | 10.5 (9.9-11.2) |
| White Cell Count | 12.9 (11.0-14.8) |
| Platelets | 207.8 (172.0-242.6) |
| International Normalised Ratio | 1.39 (1.29-1.5) |
| APTTR | 1.32 (1.3-1.4) |
| C- Reactive Protein | 135.6 (98.4-172.9) |
| Sodium | 142.7 (141.3-144.1) |
| Potassium | 4.5 (4.3-4.6) |
| Urea | 8.5 (7.0-10.1) |
| Creatinine | 104.1 (86.7-121.5) |
| Alkaline Phosphatase | 77.5 (64.9-90.2) |
| Aspartate Aminotransferase | 145.9 (88.8-203.0) |
| Bilirubin | 17.9 (10.3-25.5) |
| Albumin | 27.7 (25.6-29.9) |
| Corrected Calcium | 2.11 (2.05-2.16) |
| Phosphate | 1.2 (1.0-1.3) |
| Magnesium | 1.0 (0.93-1.1) |
| PaO_2_ | 13.7 (12.5-14.9) |
| SaO_2_ | 97.0 (96.4-97.6) |
| PaCO_2_ | 5.6 (5.2-6.0) |
| pH | 7.37 (7.35-7.38) |
| Base Excess | -1.25 (-2.3—0.2) |
| Bicarbonate | 22.9 (22.0-23.8) |
| Lactate | 1.93 (1.5-2.4) |
| Chloride | 111 (109-112) |

Table 1: Baseline laboratory values. APTTR=Activated Partial Thromboplastin Time Ratio

- 1. ***Flowchart of patients recruitment and survival within study***

Of these, 7 did not survive 10 days, 1 was transferred to another hospital, 1 withdrew from the study, 1 was discharged before day 10, and 1 was unable to have serial scans for technical reasons. (figure 1)


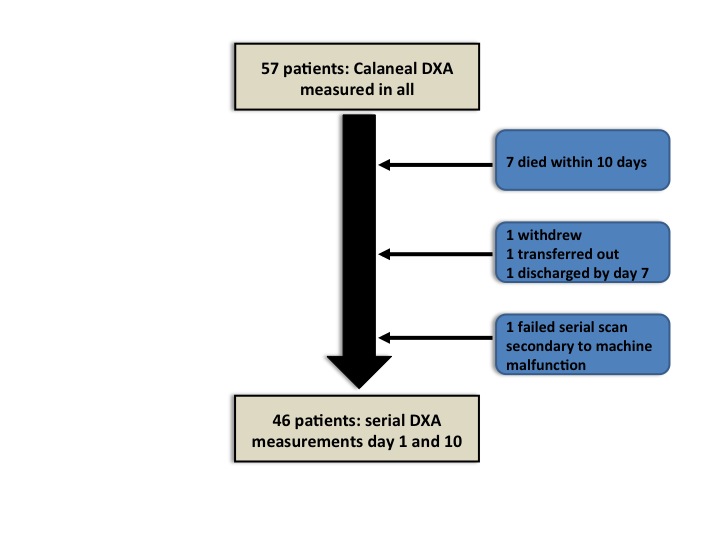

Supplement: Additional file 1: — Supplementary data. 1.1 Baseline laboratory data. 1.2 Flowchart of patient recruitment and survival within study. [file 13054_2015_892_MOESM1_ESM.docx]
